# Genetic Diversity of *Daphnia pulex* in the Middle and Lower Reaches of the Yangtze River

**DOI:** 10.1371/journal.pone.0152436

**Published:** 2016-03-25

**Authors:** Wenping Wang, Kun Zhang, Daogui Deng, Ya-Nan Zhang, Shuixiu Peng, Xiaoxue Xu

**Affiliations:** School of Life Science, Huaibei Normal University, Anhui Key Laboratory of Resource and Plant Biology, Huaibei, 235000, China; Institute of Zoology, CHINA

## Abstract

Increased human activities and environmental changes may lead to genetic diversity variations of Cladocerans in water. *Daphnia pulex* are distributed throughout the world and often regarded as a model organism. The *16S* rDNA, cytochrome c oxidase subunit I (*CO*I), and *18S* genes were used as molecular marks. The genetic diversity and phylogeny of *D*. *pulex* obtained from 10 water bodies in the middle and lower reaches of the Yangtze River were studied. For *16S* rDNA, *CO*I gene, and *18S* gene, the A+T content (65.4%, 58.4%, and 54.6%) was significantly higher than the G+C content (34.6%, 41.6% and 45.4%). This result was consistent with higher A and T contents among invertebrates. Based on the genetic distances of *16S* rDNA and *CO*I genes, the genetic differences of *D*. *pulex* from 10 water bodies located in the middle and lower reaches of the Yangtze River in China was minimal (0%–0.8% for *16S* rDNA and 0%–1.5% for *CO*I gene). However, *D*. *pulex* evolved into two branches in the phylogenetic trees, which coincided with its geographical distribution. Compared with *D*. *pulex* from other countries, the average genetic distance of *D*. *pulex* obtained from 10 water bodies in the middle and lower reaches of the Yangtze River reached 9.1%–10.5%, thereby indicating that *D*. *pulex* may have evolved into different subspecies.

## Introduction

Cladocerans are important components of the food chain in aquatic ecosystems [1]. First, these organisms can feed on algae and improve water quality [2–4]. Second, cladocerans are predated by fish as food. Cladocerans undergo parthenogenesis in suitable environments and form large populations. However, sexual reproduction of Cladocerans occurs under bad conditions and fertilized eggs are produced. *Daphnia pulex* is a cosmopolitan species that is widely distributed in inland fresh waters, particularly in eutrophic waters [5–6]. To date, frequent human activities have led to environmental differences among lakes, such as variations in nitrogen and phosphorus concentrations in the sediment in the middle and lower reaches of the Yangtze River [7]. Eutrophication and the structure of the fish population may also have affected the population dynamics of *D*. *pulex* in these lakes [8–10].

Multiple methods are available for species identification and phylogeny reconstruction of crustaceans [11–15]. The *16S* rDNA and the cytochrome c oxidase subunit I (*CO*I) and *18S* genes are more popular among these methods [15–21]. The classification of *16S* rRNA and *CO*I gene sequences were more convincing in *Daphnia*. The mitochondrial divergences of different *Daphnia* species are below 5% between North and South America [22–24] and between North America and Europe [25].

John et al. (2011) reported the gene sequences of *D*. *pulex* [26]. A few functional genes of crustaceans were widely studied [16, 20, 26–28]. Benzie (2005) described the *D*. *pulex* complex, including *D*. *pulex*, *D*. *pulicaria*, and *D*. *middendorffiana* [6]. The different *D*. *pulex* complexes are distributed worldwide, and the species was studied as a model by many investigators [28–32]. Ceresa et al. (2012) investigated the intercontinental phylogeography of the *D*. *pulex* complex by analyzing the mitochondrial NADA dehydrogenase subunit 5 and the *CO*I gene [29]. Some works in the literature showed that the genetic distance ranged from 5% to 14% for *D*. *pulex* complex [16, 29, 33]. Although the molecular phylogeny of *D*. *pulex* was extensively reported, the genetic differences of the *D*. *pulex* from China and comparison of species in China and those in other countries have not been reported.

In this study, the genetic difference among the *D*. *pulex* from 10 water bodies located in the middle and lower reaches of the Yangtze River and the genetic difference of the *D*. *pulex* between China and other countries were analyzed by amplifying and sequencing the *16S* rDNA, as well as the *CO*I and *18S* genes. Our results could become an important evidence for the global phyletic evolution of *D*. *pulex*.

## Materials and Methods

### Sampling, identification and culturing

Field collection of *Daphnia* was carried out after obtaining permission from the Ministry of Environment, and the field studies did not include endangered or protected species.

**Fig 1:**
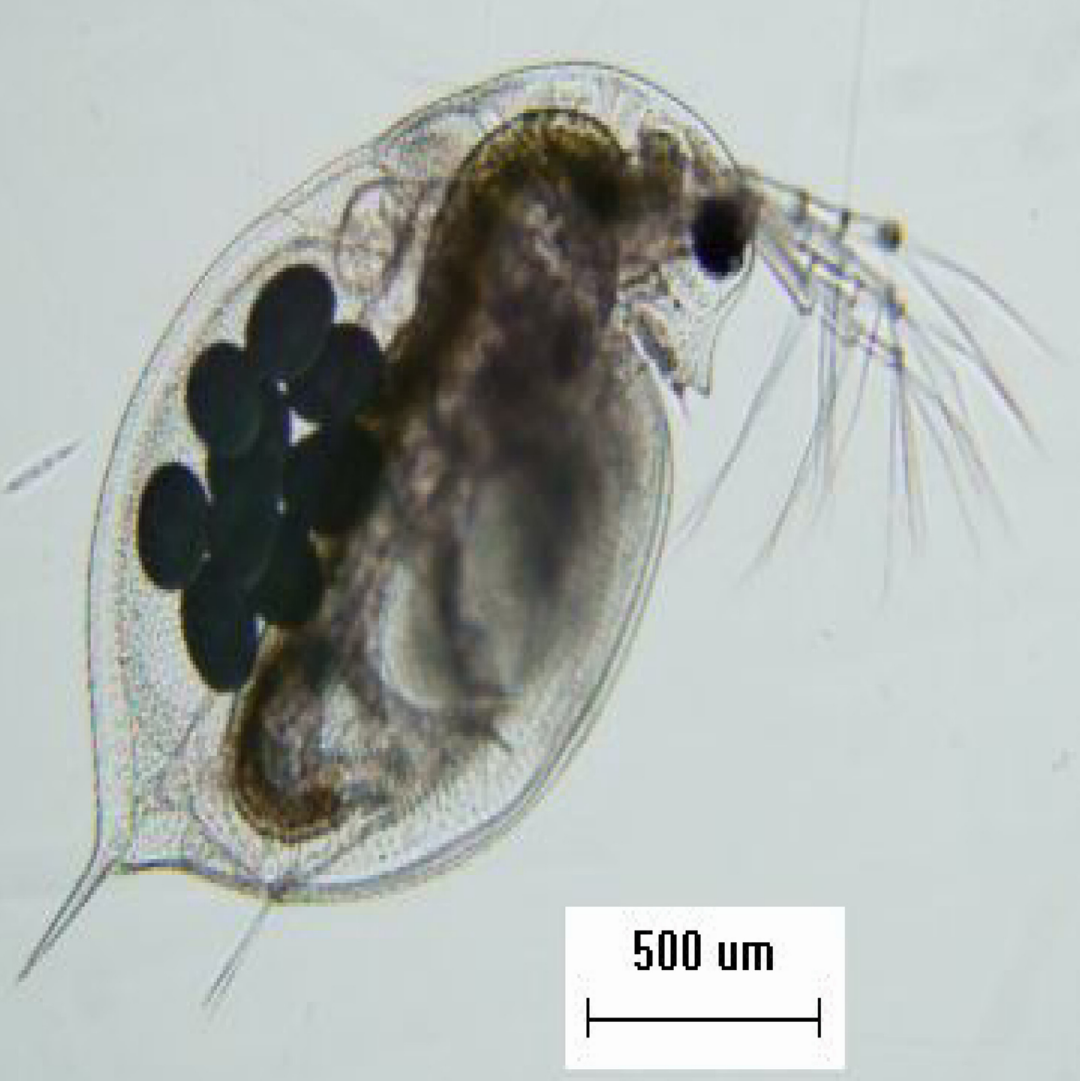
The photograph of adult *D*. *pulex* female.

The fertilized eggs of *D*. *pulex* were collected from the sediment of 10 water bodies located in the middle and lower reaches of the Yangtze River with a modified Peterson grab (Table 1). The eggs were hatched in an intelligent lighting incubator (Ningbo Saifu, China) at 25°C. *D*. *pulex* was identified morphologically (Fig 1) under the microscope (Olympus, Japan) according to the methods of Jiang and Du [5] and Benzie [6]. For each water body, four individuals of *D*. *pulex* were selected by hatching different fertilized eggs. Monoclonal organisms were cultured in an intelligent light incubator (Ningbo Saifu, China) with 12 h light:12 h dark illumination at 25°C. *Scenedesmus obliquus* was used as their food. The medium was aerated tap water over 48 h, and pH was approximately 7.

**Table 1:** Origin and number of *D*. *pulex* species in this study.

Locality	Longitude and latitude	Collection catalog numbers		
		*16S*	*CO*Ⅰ	*18S*
Donghu Lake, Hubei province	N: 30°32′46.04″ E: 114°22′31.20″	WD1WD2WD3	WD1WD2WD3	WD
Guohe River, Anhui province	N: 33°52′37.25″ E: 115°47′27.00″	BZ1BZ2	BZ1BZ2	BZ
Qianlong Lake, Anhui province	N: 33°54′21.49″ E: 116°48′55.27″	QL	QL1QL2QL3	QL
Pond in Anhui province	N: 33°38′59.33″ E: 116°57′35.21″	SZ	SZ1 SZ2	SZ
Shengjin Lake, Anhui province	N: 30°21′0.10″ E: 117°0′36.30″	SJ1 SJ2	SJ	SJ
Chaohu Lake, Anhui province	N:31°33′28.74″ E: 117°0′36.30″	CH	CH	CH1 CH2 CH3
Nanyi Lake, Anhui province	N: 31°4′27.11″ E: 118°58′40.64″	XC1XC2XC3XC4	XC1XC2XC3XC4	XC
Taihu Lake, Jiangsu province	N: 31°29′9.29″ E: 120°11′43.70″	TZ	TZ	TZ
Hongze Lake, Jiangsu province	N: 33°17′48.74″ E: 118°39′44.37″	HZ1HZ2	HZ	HZ
Pond in Shanghai city	N: 31°13′48.02″ E: 121°24′16.20"	SH1 SH2	SH1 SH2 SH3	SH

Note: Four different individuals were collected from each waterbody, but only one individual was chosen for each sequence. N indicates the North latitude, and E indicates East longitude.

### DNA extraction, amplification, and sequencing

Young *D*. *pulex* hatched from the fertilized eggs became adults and became pregnant after roughly 7 days at 25°C. An adult individual was selected. Genomic DNA of *D*. *pulex* was extracted by the TIANamp Micro DNA Kit (Tiangen, Beijing). Each *D*. *pulex* body was crushed with a sterile 10 μL tip before extraction because the chitin carapace of *D*. *pulex* could hinder the digestion of internal organs by proteinase K. The concentration of DNA extraction was measured by the Spectrophotometer (Biofuture, England). The concentrations of DNA were 65–85 ng/μL and the A280/A260 was 2.3.

The mitochondrial *16S* rDNA was amplified with the L2510 (5′-CGCCTGTTTAACAAAAACAT-3′) and H3059 (5′-CCGGTCTGAACTCAGATCATGT-3′) primers [34]. The mitochondrial *CO*I gene was amplified with the *CO*IF (5′-AYCAATCATAAGGACYATTGGRAC-3′) and *CO*IR (5′-KGTGATWCCNACHGCTCAKAC-3′) primers from Xu et al. [35]. The nuclear *18S* gene was amplified with the *18S*-F (5′-AACCTGGTTGATCCTGCCAGT-3′) and *18S*-R (5′-TGATCCTTCTGCAGGTTCACCTAC-3′) primers from Huang et al. [19].

To validate the predicted sequences of chemosensory genes, the PCR (Eppendorff, Germany) products obtained from genomic DNA of adult *D*. *pulex* were purified using the AxyPrep TM PCR Cleanup Kit (Axygen) and sub-cloned into a T/A plasmid using the pEASY-T3 cloning vector system (TransGen, China) following the manufacturer’s instructions. The plasmid DNA was used to transform to Trans1-T1 competent cells. The positive clones were checked by PCR and sequenced by GenScript (Nanjing, China).

The 25 μL PCR reaction contained 1.0 μL of genomic DNA, 14.75 μL of double-distilled H_2_O, 2.5 μL of 10× LA-Taq Buffer II, 4.0 μL of dNTPs (2.5 mM) (Shanghai Shenggong, China), 0.5 μL of Mg^2+^ (25 mM), 1.0 μL of each primer (10 mM) (Shanghai Shenggong, China), and 0.25 μL of DNA polymerase TaKaRa-LA-Taq (5 U/μL) (Clontech, USA).

The conditions of the *16S* rDNA amplification included an initial denaturing step of 3 min at 94°C, 35 cycles of 45 s at 94°C, 45 s at 50°C, and 55 s at 72°C, and a final extension of 72°C for 10 min. The conditions of the *CO*I gene amplification included an initial denaturing step of 1 min at 94°C, 35 cycles of 40 s at 94°C, 40 s at 45°C, and 1 min at 72°C, and a final extension of 72°C for 10 min. The conditions of the *18S* gene amplification included two cycles of 30 s at 94°C, 45 s at 60°C, and 45 s at 72°C, followed by five cycles of 30 s at 93°C, 45 s at 55°C, and 45 s at 72°C, and a final 35 cycles of 30 s at 93°C, 30 s at 50°C, and 3 min at 72°C.

### Analytical procedure

According to the peak in SeqMan, the bidirectional sequencing of the nucleotide sequence was proofread by DNAStar to remove unreliable bases. The percentage of the detected sequence differences was obtained.

For each water body in the middle and lower reaches of Yangtze River, the sequences of the four *D*. *pulex* individuals were obtained. Unreliable bases were removed by SeqMan (DNAStar). A total of 517–539 valid bases for *16S* rDNA, 522–527 valid bases for the *CO*I gene, and 2335–2344 valid bases for the *18S* genes were detected (Table 1). Other sequences that were used for analysis were downloaded from GenBank (Tables 2–4). In this study, the standard of the selected sequences was the similarity of the homologous sequence (over 80%) compared with the sequences from Genbank.

**Table 2:** *16S* rDNA sequences of *Daphnia* and *Bosmina* from GenBank.

Species	Code in the study	GenBank accession number	Collection location	Reference
*Daphnia pulex*	KF64	KF993364	China	Xu et al. [35]
*D*. *pulex*	KF63	KF993363	China	Xu et al. [35]
*D*. *pulex*	AF17	AF117817	Canada	Crease et al. [40]
*D*. *pulex*	JN07	JN874607	Russia	Zuykova et al. [42]
*D*. *pulex*	JN06	JN874606	Russia	Zuykova et al. [42]
*D*. *pulex*	JN05	JN874605	Russia	Zuykova et al. [42]
*D*. *pulex*	GQ75	GQ343275	Canada	Briski et al. [41]
*D*. *parvula*	GQ64	GQ343264	Canada	Briski et al. [41]
*D*. *parvula*	GQ65	GQ343265	Canada	Briski et al. [41]
*D*. *parvula*	GQ66	GQ343266	Canada	Briski et al. [41]
*D*. *parvula*	GQ67	GQ343267	Canada	Briski et al. [41]
*D*. *parvula*	GQ71	GQ343271	Canada	Briski et al. [41]
*D*. *parvula*	FJ73	FJ427473	Canada	Adamowicz et al. [33]
*D*. *cf*. *parvula* sp.	FJ74	FJ427474	Canada	Adamowicz et al. [33]
*D*. *obtusa* group sp.	FJ71	FJ427471	Canada	Adamowicz et al. [33]
*D*. *obtusa*	FJ66	FJ427466	Canada	Adamowicz et al. [33]
*D*. *obtusa* group sp.	FJ70	FJ427470	Canada	Adamowicz et al. [33]
*D*. *obtusa* group sp.	FJ67	FJ427467	Canada	Adamowicz et al. [33]
*D*. *magna*	*D*. *magna*	AY921452	USA	Colbourne et al. [46]
*Bosmina* sp.	*Bosmina*	EU650743	USA	Kotov et al. [18]

Note: *D*. *pulex* (GenBank accessions: KF993364 and KF993363) were obtained from Lake Chaohu in China.

**Table 3:** *CO*I gene sequences of *Daphnia* and *Ceriodaphnia* from GenBank.

Species	Code in the study	GenBank accession	Collection location	Reference
*Daphnia*. *pulex*	KJ74	KJ461674	China	Geng et al. [47]
*D*. *pulex*	KF72	KF993372	China	Xu et al. [35]
*D*. *pulex*	KF71	KF993371	China	Xu et al. [35]
*D*. *cf*. *pulex*	GU92	GU595192	Japan	Kotov et al. [43]
*D*. *cf*. *pulex*	GU90	GU595190	Japan	Kotov et al. [43]
*D*. *jollyi*	*D*. *jollyi*	AF308969	Canada	Hebert et al. (2000)
*Ceriodaphnia cf*. *reticulata*	*C*.*cf*.*reticulata*	KC617252	Mexico	Prosser et al. [48]

Note: *D*. *pulex* (GenBank accession: KJ461674, KF993372, and KF993371) were obtained from Lake Chaohu in China.

**Table 4:** *18S* gene sequences of *Daphnia* and *Ceriodaphni*a from GenBank.

Species	Code in the study	GenBank accession	Collection location	Reference
*D*. *pulex*	KJ027	KJ775027	China	Huang et al. [19]
*D*. *pulex*	AF011	AF014011	Canada	Crease et al. (1997)
*D*. *obtusa*	AY600	AY887600	Canada	McTaggart et al. [49]
*D*. *obtusa*	AY601	AY887601	Canada	McTaggart et al. [49]
*D*. *obtusa*	AY604	AY887604	Canada	McTaggart et al. [49]
*D*. *obtusa*	AY608	AY887608	Canada	McTaggart et al. [49]
*D*. *obtusa*	AY611	AY887611	Canada	McTaggart et al. [49]
*D*. *obtusa*	AY612	AY887612	Canada	McTaggart et al. [49]
*D*. *obtusa*	AY614	AY887614	Canada	McTaggart et al. [49]
*D*. *obtusa*	AY624	AY887624	Canada	McTaggart et al. [49]
*D*. *obtusa*	AY630	AY887630	Canada	McTaggart et al. [49]
*D*. *obtusa*	AY642	AY887642	Canada	McTaggart et al. [49]
*D*. *obtusa*	AY545	AY887545	Canada	McTaggart et al. [49]
*D*. *obtusa*	AY547	AY887547	Canada	McTaggart et al. [49]
*D*. *obtusa*	AY552	AY887552	Canada	McTaggart et al. [49]
*D*. *obtusa*	AY562	AY887562	Canada	McTaggart et al. [49]
*D*. *obtusa*	AY565	AY887565	Canada	McTaggart et al. [49]
*D*. *obtusa*	AY568	AY887568	Canada	McTaggart et al. [49]
*D*. *obtusa*	AY577	AY887577	Canada	McTaggart et al. [49]
*D*. *obtusa*	AY578	AY887578	Canada	McTaggart et al. [49]
*D*. *obtusa*	AY580	AY887580	Canada	McTaggart et al. [49]
*D*. *obtusa*	AY582	AY887582	Canada	McTaggart et al. [49]
*D*. *obtusa*	AY583	AY887583	Canada	McTaggart et al. [49]
*D*. *obtusa*	AY598	AY887598	Canada	McTaggart et al. [49]
*D*. *magna*	*D*. *magna*	AM490278	Belgium	Van Damme et al. [50]
*Ceriodaphnia dubia*	*C*. *dubia*	AF144208	USA	Spears et al. [51]

Note: *D*. *pulex* (GenBank accession: KJ775027) was obtained from Zhejiang province in China.

Multiple sequence alignment was performed with CLUSTALX (ref.). DNAspV5 (ref.) was used to analyze the variation of sites among the sequences. The conversion/transversion and the genetic distance of interspecies were calculated with MEGA 6.0 (ref.). The genetic distances among sequences were calculated by the Kimura two-parameter model with 1,000 bootstraps. The maximum likelihood (ML) analysis, which used the GTR+G+I evolutionary model indicated by Modeltest version 3.7, was performed with MEGA 6.0 (ref.) and bootstrap resampled 1,000 times. In addition, we constructed phylogenetic trees via Bayesian inference in MrBayes 3.1.2 (ref.). This program was run for 10,000,000 generations, and sampling from the chain was performed every 10,000 generations. Initially, 25% of the trees were discarded as burn-in, and the 50% majority rule consensus tree was constructed from the remaining Bayesian trees after the posterior probability values for each node were calculated. To better reveal the genetic difference of *D*. *pulex*, the suitable outgroups were employed to construct phylogenetic trees. For *16S* rDNA, *D*. *magna* (AY921452) and *Bosmina* sp. (EU650743) were used as outgroups. For *CO*I gene, *D*. *jollyi* (AF308969) and *Ceriodaphnia cf*. *reticulata* (KC617252) were used as outgroups. For *18S* gene, *D*. *magna* (AM490278) and *C*. *dubia* (AF144208) were used as outgroups. Analysis of molecular variance (AMOVA) test was conducted by using Arlequin 3.5 [36].

## Results

### Genetic diversity of *D*. *pulex* from the middle and lower reaches of Yangtze River based on *16S* rDNA

**Fig 2:**
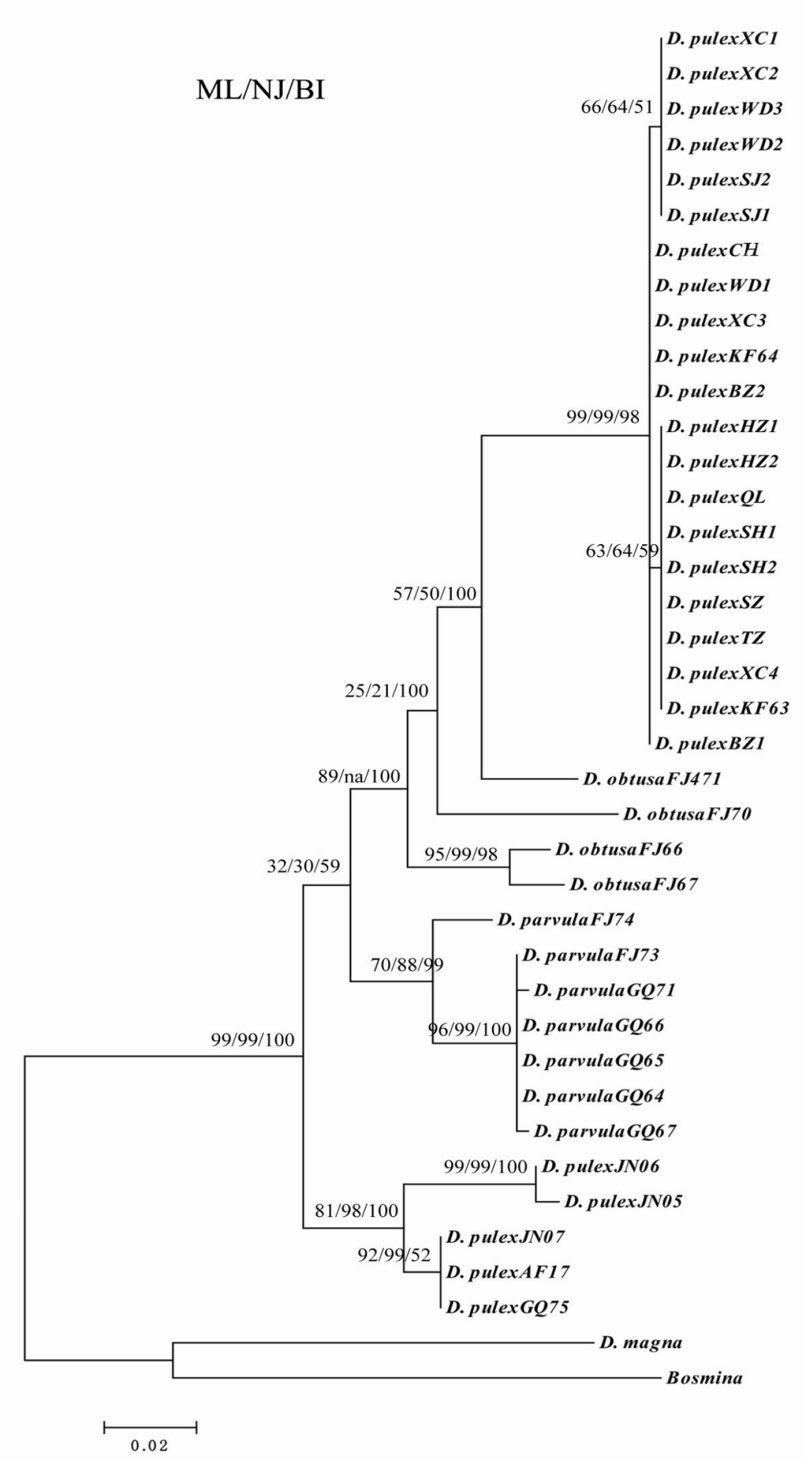
The phylogeny of *D*. *pulex* inferred from *16S* rDNA sequences as a consensus tree formed from trees constructed using maximum likelihood (ML), and neighbor-joining (NJ), Bayesian inference (BI) methods.

The alignment of the 37 *16S* rDNA sequences identified 403 conserved sites, including 334 invariable sites, 69 variable sites, 9 single sites, and 60 parsimony-informative sites. Among the *16S* rDNA sequences of the *D*. *pulex* from 10 water bodies located in the middle and lower reaches of Yangtze River, the average A, T/U, C, and G content was 32.6%, 32.8%, 13.6%, and 21.0%, respectively. The A+T content (65.4%) was significantly higher than the G+C content (34.6%). The overall transition/transversion ratio was 1.09. The genetic distances between sequences were calculated by the Kimura 2-parameter distance (0%–9.8%) and maximum likelihood estimate (0%–11.5%). The phylogenetic trees produced highly congruent tree topologies (Fig 2). The main divergences in the ML tree were in accordance with those of the MrBayes and NJ trees (Fig 2). In the phylogenetic trees, the branches represented 99% support for the presumed biological species of *D*. *pulex*. *D*. *pulex* from 10 water bodies located in the middle and lower of the Yangtze River evolved into two branches in the NJ tree. One branch included *D*. *pulex* from Lake Donghu in Hubei Province, as well as Lake Shengjin, Lake Nanyi, Lake Chaohu, and Guohe River in Anhui Province. The other branch included *D*. *pulex* from a pond in Shanghai City, Lake Taihu, and Lake Hongze in Jiangsu Province, as well as Lake Qianlong, a pond, Lake Chaohu, and Lake Nanyi in Anhui Province. *D*. *pulex* from Lake Chaohu and Lake Nanyi was present in both branches, which coincided with its geographical locations. In addition, the *D*. *pulex* from 10 water bodies located in the middle and lower reaches of the Yangtze River and the *D*. *pulex* from abroad were clustered in two distant branches (Fig 2).

### Genetic diversity of *D*. *pulex* from the middle and lower reaches of Yangtze River based on the *CO*I gene

**Fig 3:**
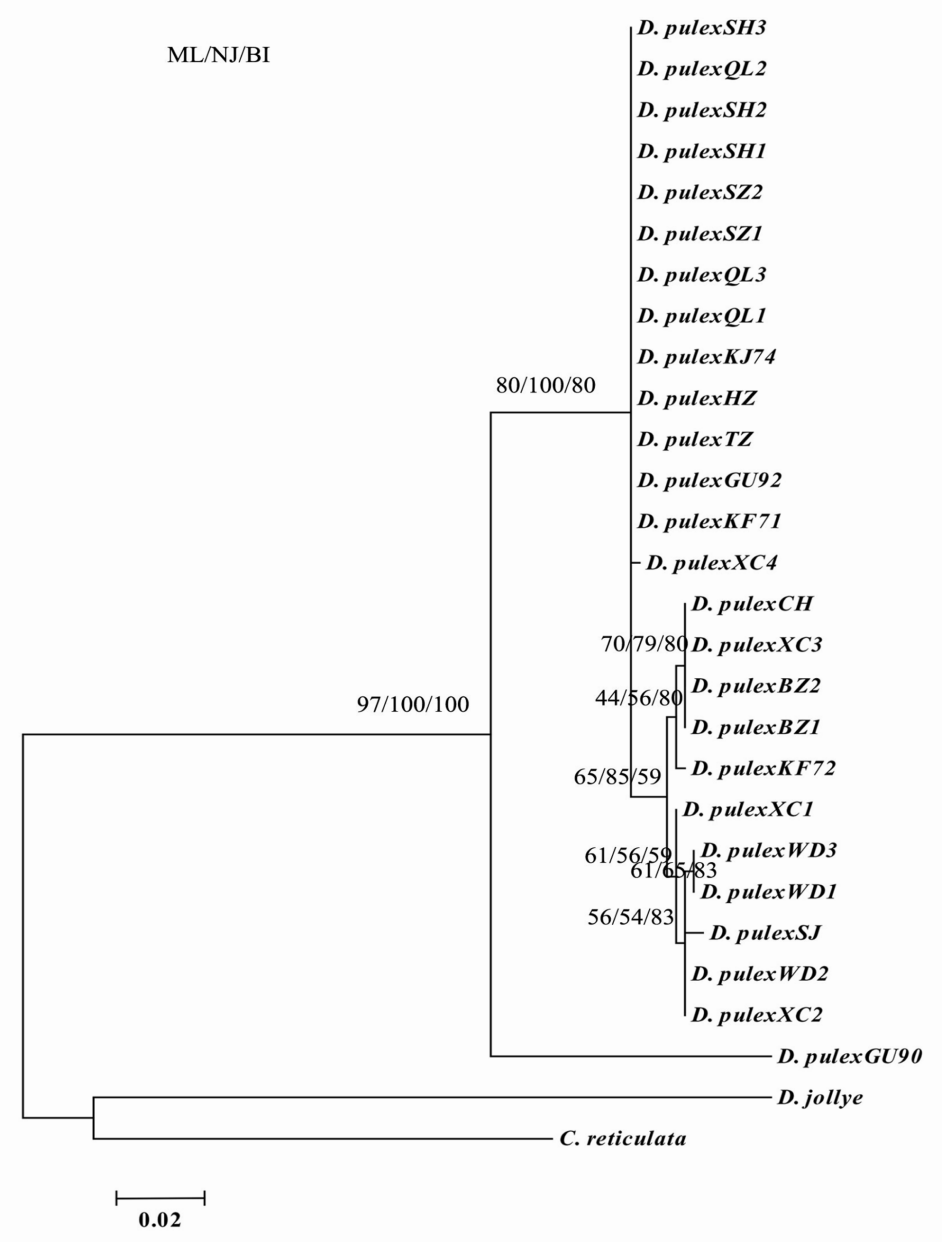
The phylogeny of *D*. *pulex* inferred from mitochondria cytochrome c oxidase subunit I (*CO*I) sequences as a consensus tree formed from trees constructed using maximum likelihood (ML), and neighbor-joining (NJ), Bayesian inference (BI) methods.

The alignment of 26 *CO*I sequences identified 487 conserved sites, including 433 invariable sites, 54 variable sites, 9 single sites, and 45 parsimony-informative sites. Among the *CO*I sequences of the *D*. *pulex* from 10 water bodies located in the middle and lower reaches of Yangtze River, the average A, T/U, C, and G content was 23.5%, 34.9%, 20.1%, and 21.5%, respectively. The A+T content (58.4%) was significantly higher than the G+C content (41.6%). The overall transition/transversion ratio was eight. The genetic distances between sequences were calculated by the Kimura two-parameter distance (0%–11.3%) and maximum likelihood estimate (0%–11.4%). The main divergence in the ML tree was in accordance with that of the MrBayes tree and NJ tree (Fig 3). In the phylogenetic trees, the branches represented 100% support for the presumed biological species of *D*. *pulex*. The *D*. *pulex* from 10 water bodies located in the middle and lower reaches of Yangtze River diverged into two branches in the phylogenetic trees, which was consistent with the results of *16S* rDNA sequence analysis. In addition, the *D*. *pulex* (GU595190) from Japan and the *D*. *pulex* from 10 water bodies located in the middle and lower reaches of Yangtze River were evidently different, with an average genetic distance of 10.5%.

### Genetic diversity of *D*. *pulex* from the middle and lower reaches of Yangtze River based on the *18S* gene

**Fig 4:**
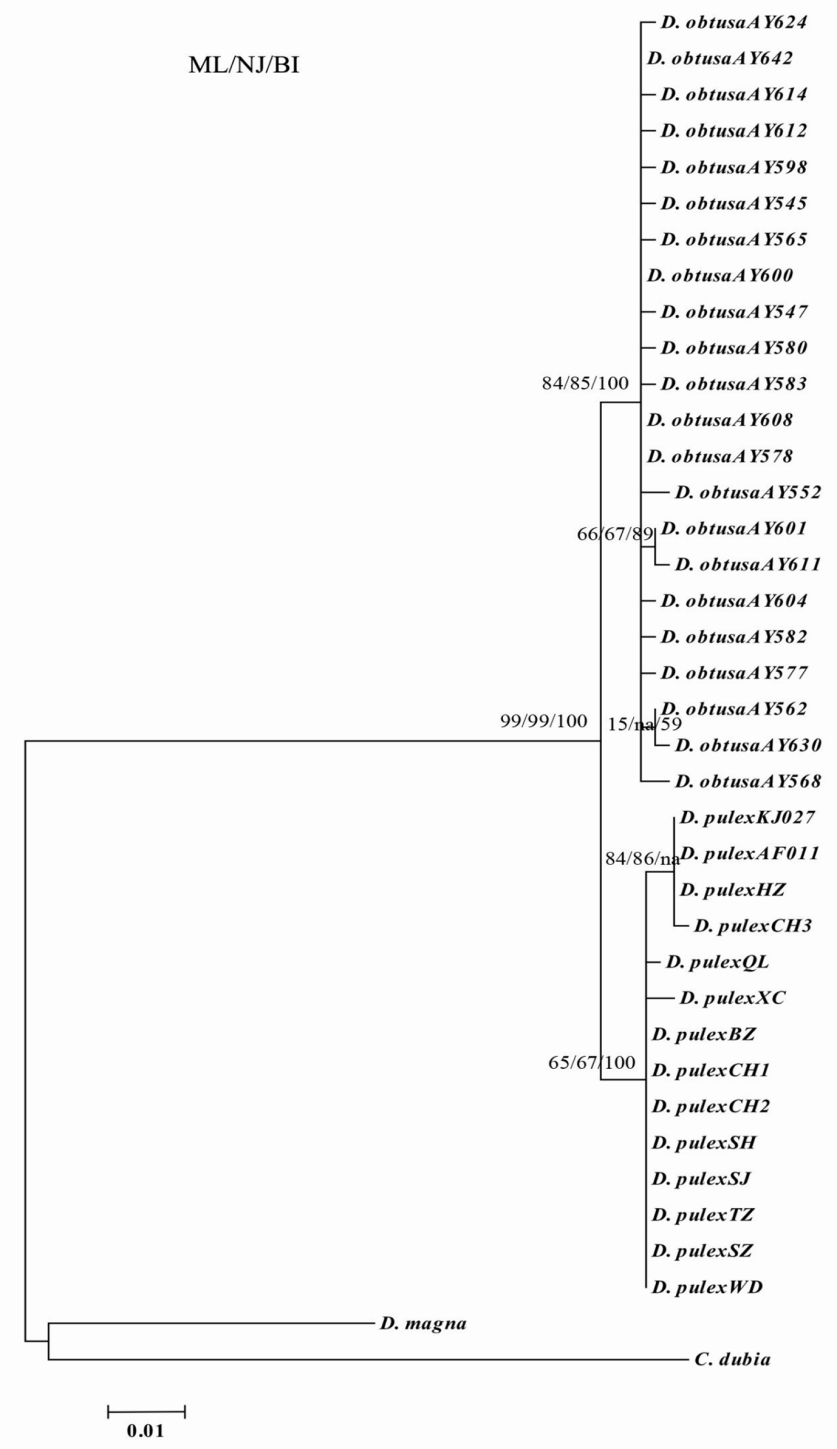
The phylogeny of *D*. *pulex* inferred from *18S* gene sequences as a consensus tree formed from trees constructed using maximum likelihood (ML), and neighbor-joining (NJ), Bayesian inference (BI) methods.

The alignment results of 36 *18S* gene sequences identified 1963 conserved sites, including 1932 invariable sites, 31 variable sites, 20 single sites, and 11 parsimony-informative sites. Among the sequences of the *18S* gene for the *D*. *pulex* from 10 water bodies located in the middle and lower reaches of Yangtze River, the average A, T/U, C, and G contents were 20.5%, 24.9%, 24.3%, and 30.3%, respectively. The A+T content (54.6%) was significantly higher than the G+C content (45.4%). The overall transition/transversion ratio was 2.5. The genetic distances between sequences were calculated by the Kimura two-parameter distance (0%–2.0%) and the maximum likelihood (0%–1.3%). The main divergence in the ML tree was in accordance with that of the MrBayes and NJ trees. The phylogenetic trees produced highly congruent tree topologies (Fig 4). In the phylogenetic trees, the branches represented 99% support for a presumed biological species of *D*. *pulex*. The *D*. *pulex* from Lake Chaohu in Anhui province (CH3), Lake Hongze in Jiangsu province, Hangzhou City in Zhejiang province (KJ775027), and Canada (AF014011) belonged to the same branch, whereas smaller differences were observed with the other *D*. *pulex* individuals. The average genetic distance was 0.45%–0.64%.

### Tests on the genetic difference of *D*. *pulex* from the middle and lower reaches of Yangtze River

**Fig 5:**
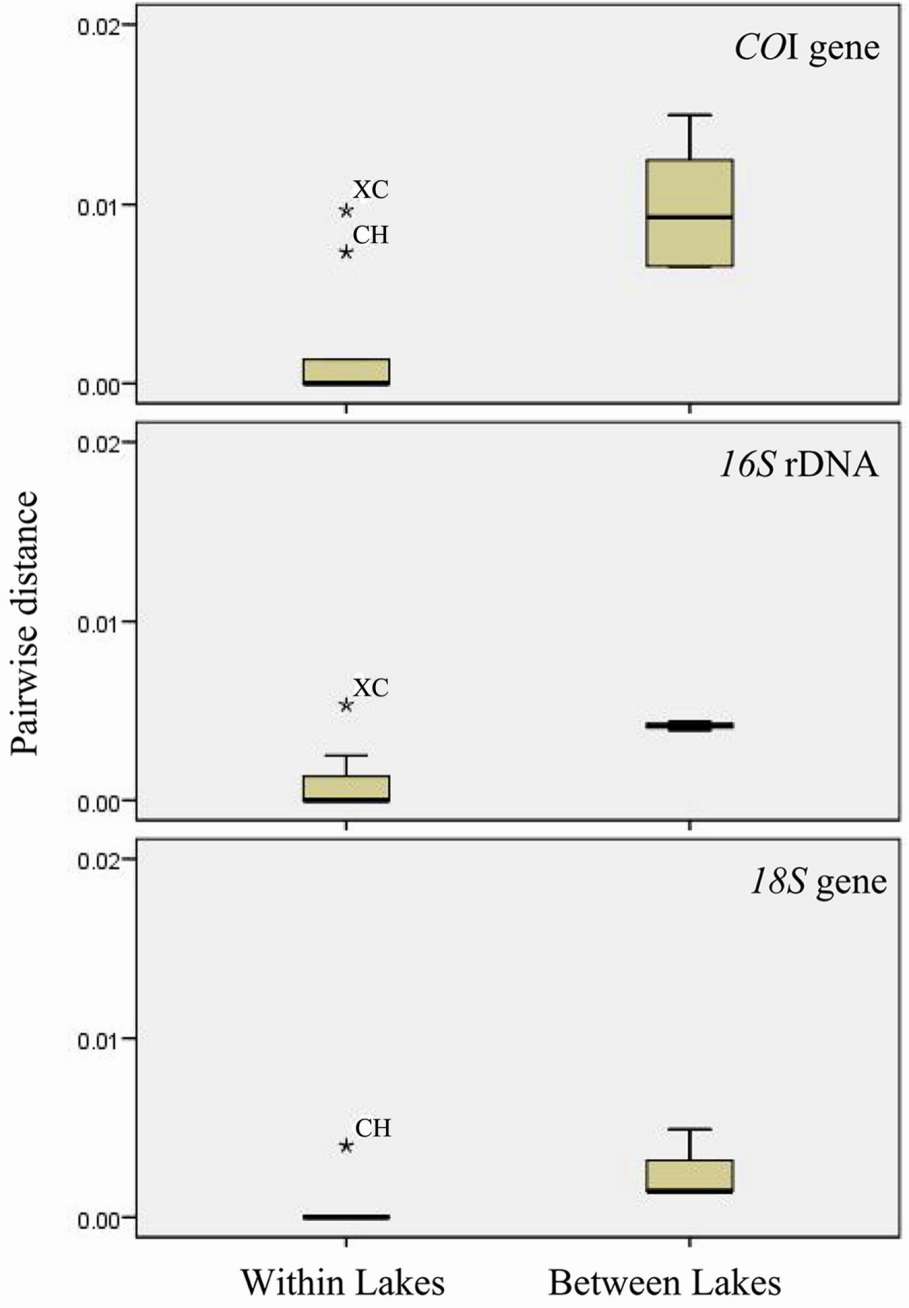
Box diagram of *D*. *pulex* genetic diversity within-lakes and between-lakes (XC: Lake Nanyi; CH: Lake Chaohu).

Based on the sequences of *16S* rDNA, *CO*I gene, and *18S* gene, the genetic differences of *D*. *pulex* were analyzed within lakes and between lakes in the middle and lower reaches of Yangtze River. Mann-Whitney Test showed that the genetic differences of the *D*. *pulex* between within-lakes and between-lakes were significant (*CO*I gene: Z = -3.172, *P* = 0.002; *16S* rDNA: Z = -3.096, *P* = 0.002; *18S* gene: Z = -3.378, *P* = 0.001). Two-Sample Kolmogorov-Smirnov test showed the significant differences in both within-lakes and between-lakes (*CO*I gene: Z = 1.789, *P* = 0.003; *16S* rDNA: Z = 2.012, *P* = 0.001; *18S* gene: Z = 2.012, *P* = 0.001). The box diagram of *D*. *pulex* genetic diversity also demonstrated significant differences between within-lakes and between-lakes based on the sequences of *16S* rDNA, *CO*I gene, and *18S* gene (Fig 5), which indicate that the genetic structure of *D*. *pulex* exhibiting differentiation among lakes.

Within lakes, the genetic difference of *D*. *pulex* from Lake Chaohu (CH) was bigger than that of other lakes based on the sequences of *CO*I gene and *18S* gene, and the genetic difference of *D*. *pulex* from Lake Nanyi (XC) was bigger than that of other lakes based on the sequences of *CO*I gene and *16S* rDNA (Fig 5).

### Analysis of molecular variance

Based on the sequences of *16S* rDNA, *CO*I gene and *18S* gene, the Molecular Variance of *D*. *pulex* were analyzed to calculate the population genetics for each site in the middle and lower reaches of Yangtze River. The result suggested that the pairwise differences were greater among populations than within populations based on the sequences of *16S* rDNA, *CO*I gene and *18S* gene (Table 5). The AMOVA test showed that there were significant differences between two groups (middle reach and lower reach) based on *16S* rDNA and *CO*I gene (Table 6).

**Table 5:** Analysis of molecular variance (AMOVA) based on the *16S* rDNA, *CO*I gene and *18S* gene sequences of *D*. *pulex* in the middle and lower reaches of Yangtze River.

	Source of variation	Degrees of freedom	Sum of squares	Variance components	Variation (%)	*p*-value	FST
*16S* rDNA	Among population	9	158.425	3.63403 Va	59.23		
*16S* rDNA	Within population	30	92.000	2.56667 Vb	40.77		
*16S* rDNA	Total	39	250.425	6.20069		0.000	0.59234
*CO*I gene	Among population	9	124.850	2.90139 Va	56.14		
*CO*I gene	Within population	30	68.000	2.26667 Vb	43.86		
*CO*I gene	Total	39	192.850	5.16806		0.000	0.56141
*18S* gene	Among population	9	300.275	8.12222 Va	90.27		
*18S* gene	Within population	30	26.250	0.87500 Vb	9.73		
*18S* gene	Total	39	326.525	8.99722		0.000	0.90275

Note: populations were evaluated as a single group. Each lake was as a population.

**Table 6:** Analysis of molecular variance (AMOVA) based on the *16S* rDNA and *CO*I gene sequences of *D*. *pulex* about the two groups (middle reach vs. lower reach) in the Yangtze River.

	Source of variation	Degrees of freedom	Sum of squares	Variance components	Variation (%)	FSC/ FST	*p*-value
*16S* rDNA	Among groups	1	34.240	1.29190 Va	20.22		
*16S* rDNA	Among populations within groups	6	89.167	3.25434 Vb	50.93		
*16S* rDNA	Within populations	24	44.250	1.84375 Vc	28.85		
*16S* rDNA	Total	31	167.656	6.38999		0.63834/ 0.71146	0.000/ 0.000
*CO*I gene	Among groups	1	48.385	2.79421 Va	54.88		
*CO*I gene	Among populations within groups	6	38.833	1.39149 Vb	27.33		
*CO*I gene	Within populations	24	21.750	0.90625 Vc	17.80		
*CO*I gene	Total	31	108.969	5.09196		0.60559/ 0.82202	0.000/ 0.000

Note: populations were evaluated as two groups (middle reach vs. lower reach) except Lake Chaohu and Lake Nanyi. Each lake was regarded as a population.

## Discussion

For *16S* rDNA and *CO*I gene sequences of *D*. *pulex* from 10 water bodies located in the middle and lower reaches of Yangtze river, the A+T content (65.4% and 58.4%, respectively) was significantly higher than the G+C content (34.6% and 41.6%, respectively). Those results were consistent with the higher A and T contents among invertebrates [37–38]. Moreover, the overall transition/transversion bias of *D*. *pulex* based on *CO*I gene (8) was obviously higher than those based on the *16S* rDNA (1.09) and *18S* gene (2.5).

Based on the genetic variation of the *16S* rDNA and *CO*I genes, the *D*. *pulex* from 10 water bodies located in the middle and lower reaches of the Yangtze River evolved into two branches, as shown in the phylogenetic trees. One branch included the *D*. *pulex* from Lake Donghu in Hubei Province, as well as Lake Shengjin, Lake Nanyi, Lake Chaohu, and Guohe River in Anhui Province. The other branch included *D*. *pulex* from a pond in Shanghai City, Lake Taihu and Lake Hongze in Jiangsu Province, as well as Lake Qianlong, a pond, Lake Chaohu, and Lake Nanyi in Anhui Province. The *D*. *pulex* from Lake Chaohu and Lake Nanyi in Anhui province were present in both branches, which coincided with its geographical distribution in the middle and lower reaches of the Yangtze River. Based on the sequences of *16S* rDNA, *CO*I gene and *18S* gene of *D*. *pulex*, the AMOVA test also showed that there all were greater genetic differences among lakes than within lakes in the middle and lower reaches of the Yangtze River. And significant genetic differences between two groups (middle reach and lower reach) were showed based on *16S* rDNA and *CO*I gene of *D*. *pulex*. Then the genetic distances of *D*. *pulex* from 10 water bodies located in the middle and lower reaches of Yangtze River showed minimal divergence based on *16S* rDNA (0%–1.0%), *CO*I gene (0%–1.7%), and *18S* gene (0%–0.9%), and all those differences were within the scope(<5%) of species [16, 33, 39]. These findings implied that the *D*. *pulex* from the lakes located in the middle and lower reaches of Yangtze River region should belong to the same species. In addition to further geographical distance, other environmental conditions, such as different climate, altitude, and fishery in the middle and lower reaches of the Yangtze River, may be important factors to the evolution of *D*. *pulex*.

Compared with the *D*. *pulex* from Canada (AF117817, GQ343275) [40, 41] and Russia (JN874605, JN874606, and JN874607) [42], the genetic distances of *D*. *pulex* from 10 water bodies located in the middle and lower reaches of the Yangtze River reached 9.1%–9.6% based on *16S* rDNA sequence. The genetic differences was obviously beyond the scope of a species (<5%) [16, 33, 39], and it indicated the presence of subspecies. Long-term geographic isolation may be the main reason for the evolution of the *D*. *pulex* in China and other countries. In addition, the average genetic distance between the *D*. *pulex* in Japan (GU595190) and the *D*. *pulex* in China reached 10.5% based on the *CO*I gene sequence. The genetic distance was in the scope of the *Daphnia* complex (5%–14%). Thus, compared with the *D*. *pulex* (GU595190) in Japan, the *D*. *pulex* from China should belong to different subspecies or the *D*. *pulex* complexes [6, 16, 33, 43]. On the other hand, the genetic distance of the *D*. *pulex* (GU595192) in Japan and in China was below 5%. We speculated that the *D*. *pulex* had same ancestor and evolved to different directions by natural selection in Japan and China. Although the average genetic distances of the *D*. *pulex* from Canada (AF014011) and from China were small (0.45%–0.64%) based on the *18S* gene sequence, the difference was evident. In general, the evolutionary divergences of the *D*. *pulex* among different lakes located in the middle and lower reaches of the Yangtze River were minimal. However, the evolutionary divergence was relatively high compared with that of other countries. The global molecular phylogeny of *D*. *pulex* needs to be further studied and discussed.

The intercontinental phylogeny of the *D*. *pulex* complex is extremely complicated. Based on the sequences of the mitochondrial dehydrogenase NADH 5 subunit and *CO*I genes of 398 *D*. *pulex* individuals from five continents, Crease et al. (2012) concluded that 11 lineages of the *D*. *pulex* complex can be observed worldwide [29]. By studying the *D*. *pulex* complex from 12 Bolivian high-altitude lakes, the *D*. *pulicaria* group in North America was found to originate in South America, whereas these South American water fleas originated through reciprocal hybridization between different sexually reproducing parental lineages [44]. In the present study, based on the *16S* rDNA sequence, the average genetic distances of the *D*. *pulex* from China and the *D*. *parvula* and the *D*. *obtusa* from Canada were 7.3% and 8.2%, respectively. Their differences belong to the scope of the *D*. *pulex* complex (5%-14%) [16, 29–33, 45]. Benzie (2005) hypothesized that the main factor that led to the formation of species complexes between the *D*. *pulex*, *D*. *pulicaria*, and *D*. *middendorffiana* was their long-term coexistence in the same habitat, which resulted in the occurrence of interspecies complexes [6,16]. In the middle and lower reaches of the Yangtze River in China, the coexistence of *D*. *pulex*, *D*. *galeata*, and *D*. *similoides* was common in some lakes (e.g. Lake Donghu, Lake Taihu, and Lake Chaohu) [8–10]. Thus, the existence of species complexes among *Daphnia* species in these Chinese lakes was possible, and further investigation is needed.
